# Pulmonary function evaluation after hospital discharge of patients with severe COVID-19

**DOI:** 10.6061/clinics/2021/e2848

**Published:** 2021-06-23

**Authors:** Jessica Polese, Larissa Sant’Ana, Isac Ribeiro Moulaz, Izabella Cardoso Lara, Julia Muniz Bernardi, Marina Deorce de Lima, Elaína Aparecida Silva Turini, Gabriel Carnieli Silveira, Silvana Duarte, José Geraldo Mill

**Affiliations:** I Departamento de Pneumologia, Universidade Federal do Espirito Santo, Vitoria, ES, BR; II Universidade Federal do Espirito Santo, Vitoria, ES, BR; III Departamento de Ciencias Fisiologicas, Centro de Ciencias da Saude, Universidade Federal do Espirito Santo, Vitoria, ES, BR

**Keywords:** SARS-CoV-2, COVID-19, Pulmonary Function Test, Symptoms

## Abstract

**OBJECTIVES:**

Coronavirus disease 2019 (COVID-19) may be associated with prolonged symptoms and post-recovery health impairment. This study aimed to evaluate the persistence of symptoms, lung function, and pulmonary diffusion for carbon monoxide (DLCO) in patients between 15 and 30 days after hospital discharge after admission for severe COVID-19.

**METHODS:**

The evaluation consisted of 1) comparative analysis between the initial symptoms and symptoms still present at the post-discharge evaluation 2) analysis of the chest images obtained during hospitalization, and 3) conducting spirometry, plethysmography, and DLCO assessment.

**RESULTS:**

Forty-one patients who were hospitalized for 16±8 days with severe COVID-19 were included. Patients were predominantly men (73%) and had a mean age of 51±14 years. The most frequent comorbidities were arterial hypertension (51%) and diabetes mellitus (37%). Pulmonary evaluation was performed a mean of 36 days after the onset of symptoms, with the most frequent persistent symptoms being dyspnea (83%) and coughing (54%). Approximately 93% of patients still had at least one symptom, and 20% had more than five symptoms. Chest imaging revealed a typical pattern of COVID-19 on X-ray (93%) and computer tomography (95%). Lung function test results showed a restrictive pattern with a reduction in forced vital capacity (FVC) in 54% of individuals, with an average FVC of 78±14%. A reduction in DLCO was observed in 79% of patients.

**CONCLUSIONS:**

We observed a high prevalence of symptoms, in addition to a significant change in lung function and DLCO, in the post-discharge assessment of patients requiring hospitalization after admission for COVID-19.

## INTRODUCTION

Coronavirus disease 2019 (COVID-19), which is caused by severe acute respiratory syndrome coronavirus 2 (SARS-CoV-2), was first reported in December 2019 in China, quickly spread to countries across five continents, and was declared a pandemic by the World Health Organization (WHO) on March 11, 2020. By the end of January 2021, more than 90 million cases have been registered across more than 100 countries, and more than 2 million deaths have occurred (1).

The symptoms of COVID-19 range from mild flu-like symptoms to respiratory system failure. The epidemiology of the infection indicates that the majority of patients develop milder forms of the disease, while 14% of those infected have a severe form, and a smaller percentage (5%) become critically ill (2). Among patients who have required hospitalization, 14.2% required care in the intensive care unit and 12.2% received mechanical ventilation, and the mortality in this group was approximately 24.5% (3). Pulmonary manifestations are the most common due to the route of entry of the virus, which uses angiotensin-converting enzyme 2 receptors present in type 2 pneumocytes, leading to a subsequent inflammatory response (4).

The demographic profile and risk factors for COVID-19 show a wide spectrum around the world, and the factors responsible for the occurrence of different clinical forms and variability of symptoms are not yet understood. Moreover, health issues that persist for more than four weeks after COVID-19 infection, known as post-COVID conditions, are still not well understood, and present a major challenge to health systems worldwide given the high number of individuals affected by the disease and who recover after varying periods of hospitalization (5).

To date, few studies have evaluated the clinical evolution and the occurrence of structural and functional post-COVID conditions in the lungs of individuals who survive the severe form of the disease, mainly because it is a new and recent disease. Initial studies described possible long-term complications of COVID-19 including cardiovascular, pulmonary, metabolic, and neuropsychiatric sequelae based on data from the severe acute respiratory syndrome (SARS) and Middle East respiratory syndrome (MERS) epidemics (6). Recent publications have described the persistence of symptoms, especially fatigue and dyspnea, approximately two months after the onset of symptoms, associated with a decrease in patients’ quality of life (7,8), as well as altered respiratory function after discharge (9,10). Consequently, it is necessary to further investigate the late repercussions of COVID-19, especially in survivors of severe forms of the disease, and its effect on quality of life. The present study aimed to evaluate pulmonary function after hospital discharge of individuals who presented with the severe COVID-19.

## METHODS

A cross-sectional study was conducted at the University Hospital Cassiano Antônio Moraes (HUCAM) of the Federal University of Espírito Santo, in Vitória/ES. The project was approved by the Research Ethics Committee of HUCAM (Protocol n° 3503292000005071), and all participants signed a consent form for inclusion in the study.

According to the WHO, the diagnosis of COVID-19 consists of SARS-CoV-2 infection confirmed using reverse transcription polymerase chain reaction or a suggestive clinical presentation and reactive serology. Patients can be categorized as follows: 1) mild disease, when there are only mild symptoms and no imaging examination suggestive of pneumonia; 2) pneumonia when symptoms and imaging tests are suggestive of pneumonia but oxygen supplementation is not necessary; 3) severe pneumonia when they have a radiological evidence of pneumonia, associated with a respiratory rate of 30 breaths per min or SpO_2_ of 93% at rest; 4) acute respiratory distress syndrome (ARDS) when the injury progresses to affect >50% of the lung on pulmonary imaging within 24-48h (11).

Patients of both sexes, aged >18 years, hospitalized with a diagnosis of COVID-19 who needed supplemental oxygen or ventilatory support and presented with a severe form of the disease, defined as either severe pneumonia or ARDS, were recruited to the study at the time of hospital discharge (September to October 2020). The patients presented a discharge summary containing what happened during hospitalization, as well as radiological examinations performed during hospitalization. Because the focus of the study was the evaluation of pulmonary function after COVID-19, patients who reported a previous history of heart disease with systemic repercussions, pneumopathy, nephropathy, or who were >70 years old were not included in this analysis.

Questionnaires and examinations were performed on the same day between 15 and 30 days after hospital discharge. The evaluation consisted of three stages:

Stage 1: Application of a standardized questionnaire to define the demographic profile, main symptoms at the onset of disease and currently, evolution during hospitalization, need for intubation, presence of self-reported comorbidities, use of medications, and weight and height to calculate body mass index (BMI).

Stage 2: Evaluation of images and reports of chest radiography and high-resolution computed tomography (HRCT) performed during hospitalization, with qualitative and quantitative data. The findings on chest radiography were described as follows: 1) classical findings for COVID-19 when it demonstrated multiple bilateral opacities, predominantly peripherally and concentrated in the lower lobes, and 2) indeterminate for COVID-19 when the images did not fit this description or presented with atypical findings. The quantification of lung lesions was also recorded on chest radiography, with the extent of lung involvement described as mild, moderate, or severe (12,13). The HRCT images were described as follows: 1) classical findings for COVID-19 when they exhibited the predominant pattern of bilateral, peripheral, and basal findings; 2) images not suggestive of COVID-19 defined as findings of lobar pneumonia, cavitations, centrilobular nodules, lymphadenopathy, and stroke (14). In addition, the extent of pulmonary involvement and presence of findings considered characteristic including ground-glass opacity, mosaic paving, peripheral consolidation, and inverted halo sign, as well as perilobular infiltrate pattern, were recorded.

Stage 3: Performing pulmonary function tests and the 6-minute walk test (6-MWT). For pulmonary evaluation, patients underwent plethysmography, measurement of the pulmonary diffusion capacity for carbon monoxide (DLCO) (Vyaire, Jaeger, Germany), and spirometry (Koko Sx, Philips, USA). Total lung capacity (TLC), expiratory reserve volume, residual volume (VR), forced expiratory volume in one second (FEV_1_), and forced vital capacity (FVC) DLCO was performed to assess gas exchange impairment. The standardization and categorization of functional changes were based on Brazilian guidelines for pulmonary function tests (15). FVC relative to the predicted value (PV) was characterized as mild dysfunction (FVC=60-80% of VP), moderate (FVC=50-59% of VP), and severe (FVC<50% of VP). Regarding TLC, a restrictive standard was considered if TLC was <80% of the PV. DLCO was considered altered if <80% of the PV. The 6-MWT was performed with a JG MORIYA model 1001 pulse oximeter; oxygen desaturation was defined as >3% drop in oxygen saturation (SpO_2_), and the distance covered was considered to be reduced when less than 400 m.

Categorical data are presented as the number of individuals and their respective percentages. Continuous variables are described as mean, median, and standard deviation.

## RESULTS

A total of 96 patients were referred for pulmonary function assessment. After exclusion, 41 patients were included in the analysis. The evaluation was performed a mean 36 days after symptom onset.

Hospitalization data were obtained through a medical report issued at hospital discharge, and the comorbidities and history prior to hospitalization were self-reported by the patient. The clinical presentation on admission was severe pneumonia in five (12%) patients and ARDS in 36 patients (88%), three of whom required orotracheal intubation and one of whom required tracheostomy. The average hospital stay was 8.6±6 days. Patients were predominantly men (73%), and the mean age was 51±11 years. The mean BMI was 30±5.4 kg/m^2^, with 17 (41%) overweight and 17 (41%) obese patients. Of the evaluated group, 30 (73%) patients reported previous comorbidities, including hypertension (51%) and diabetes mellitus (37%). In addition, pulmonary thromboembolism (PTE) occurred during hospitalization in six patients, and one patient developed deep vein thrombosis 45 days after symptom onset. The demographic data of the patients are shown in Table 1.

**Figure 1:**
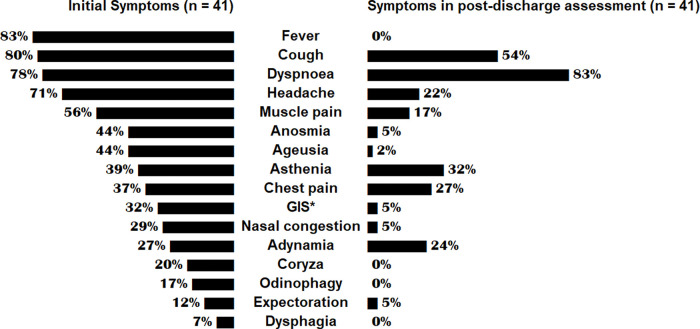
Comparison between initial symptoms and symptoms in post-discharge assessment. *GIS: Gastrointestinal symptoms.

In the post-discharge assessment, 93% of patients had at least one symptom and 20% had more than five, with dyspnea (83%) and coughing (54%) being the most frequently seen symptoms. The comparison between the symptoms at disease onset and the post-discharge assessment is shown in Figure 1.

HRCT scans from 20 patients and chest X-rays from 16 patients were analyzed. HRCT scans were compatible with severe pulmonary impairment >50% of the lung fields in eight (50%) patients. On the HRCT images, a classical pattern suggestive of COVID-19 was observed in 19 (95%) of the 20 patients, the only patient without a classical lesion with an image suggestive of pulmonary infarction had pulmonary thromboembolism diagnosed using chest angiotomography. In classical lesions, there was a predominance of diffuse and bilateral disease, the most common alterations being the ground-glass pattern, septal thickening, and consolidation. The distribution of the tomographic patterns is shown in Table 2. All individuals who underwent X-ray showed probable radiological alterations. The changes are described in Table 3.

Spirometry was performed in 41 patients, of whom 22 (54%) had decreased FVC (FVC=78.4±14%). The change in FVC was mild in 18 (44%), moderate in 3 (7.3%), and severe in 1 (2.4%) of the 41 patients. None of the patients showed changes in the FEV_1_/FVC ratio.

DLCO was performed in 14 patients; in 11 patients (79%), there was a change in diffusion. TLC was altered in seven (50%) patients, with mild changes in 36% and moderate changes in 14.3%.

The mean basal saturation before the 6-MWT was 96±2%. Hemoglobin desaturation was observed in 19 (47.5%) of the 41 patients, and 22 (55%) covered <400 m.

## DISCUSSION

This study evaluated lung function after discharge from a group of patients who developed severe pneumonia or ARDS while having COVID-19. For a better assessment of possible functional impairment following recovery from COVID-19, 55 patients with possible pre-infection pulmonary involvement were excluded from this analysis. Thus, the severity of the clinical and radiological picture that led to hospitalization, as well as the changes and symptoms presented after hospital discharge, most likely resulted from COVID-19.

The severity of the pathology in studied group can be assessed by the symptoms at the beginning of the disease and maintenance of symptoms even after an average of 16 days after hospital discharge. The assessment of pulmonary impairment on radiological images obtained during hospitalization showed impairment affecting 25-50% of the lungs in 55% of individuals and impairment affecting 50-75% of the lungs in 40% of individuals, indicating the severity of disease in hospitalized patients. The findings on HRCT were compatible with those reported in the literature and reflect severely compromised individuals (13,14,16-21).

The predominance of men (73%), high BMI, and high proportion of patients with diabetes and hypertension in the sample, are in agreement with what is described in the literature, while the average age of the sample (51 years) was slightly lower than that described in the literature for cases of severe COVID-19 (3), possibly due to the exclusion of individuals over 70 years of age.

Most of these patients at the beginning of the pandemic would have been candidates for orotracheal intubation; however, the experience gained in the management of patients has shown that it is possible to manage many of these patients with a high-flow oxygen catheter or non-invasive ventilation, which was not the recommended approach at the beginning of the pandemic. Likewise, the use of corticosteroids in COVID-19 was also not recommended at the beginning of the pandemic; however, studies have shown a reduction in the length of stay, and their use is recommended in the most severe cases with proven benefits, contributing to better progression of the condition (20). Thus, only three (7%) patients required orotracheal intubation, a number considered small when considering the severity of the cases. In addition, although the length of hospital stay was long (9±6 days), it was potentially reduced by not requiring orotracheal intubation, which reduces morbidity and hospital stay, if we consider the severity of the cases in this study.

The severity of COVID-19 seems to be due to the extent of lesions caused by the virus in vital organs, especially in the lungs, as well as the patient’s inflammatory response, resulting in a severe imbalance in the hemostasis of these individuals, which leads to a state of hypercoagulability (20). This hemostatic imbalance is responsible for the high incidence of thromboembolic events in patients with COVID-19, including venous thromboembolism (VTE) (21). The incidence of VTE in this sample was similar to that reported in another study (22). Among the cases of VTE, six (85.7%) presented with pulmonary embolism, reinforcing the hypothesis of a local mechanism that facilitates thrombosis, as already indicated in other studies (23).

Some symptoms persisted in almost all patients after hospital discharge, with 95% of patients presenting at least one symptom and 19 (51%) presenting more than five, with dyspnea (83%), coughing (54%), and chest pain (27%) the most commonly reported persistent symptoms. In most cases, the persistence of symptoms was a nuisance and hindrance to work activity, directly affecting quality of life and economic and social performance, as well as the psychological health of the patients.

In relation to pulmonary function tests, we found functional impairment in more than half of individuals, represented both by a reduction in FVC and TLC and by the low performance in the 6-MWT. A reduction in DLCO was found in 79% of the 14 patients where the test could be performed due to the availability of the equipment, which reflects changes in gas exchange, even after hospital discharge.

Case studies of ARDS secondary to SARS and MERS reported a 27% change in carbon monoxide diffusion (95% confidence interval: 15-45%) and a reduction in exercise capacity in the 6-MWT 6 months after hospital discharge, suggesting pulmonary fibrosis (23). In a study of 19 cases of severe pneumonia caused by SARS-CoV-2, an alteration in FVC was found in 2 (10.53%) and none showed an alteration in FEV/FVC; in that same study, a reduction in DLCO was found in 16 (84.21%) of the 19 cases (24). In our study, we found changes in FVC and TLC and, on average, a reduction in DLCO 16 days after hospital discharge, raising the possibility that COVID-19 could develop sequelae such as pulmonary fibrosis. As demonstrated by the recent literature, there is evidence of diffuse alveolar damage, which raises the possibility of interstitial pulmonary fibrosis in patients with COVID-19 (25); however, more detailed studies are needed to test this hypothesis.

Studies still in progress demonstrate a high possibility of late sequelae of SARS-CoV-2 infection (26). However, these late pulmonary sequelae have not yet been established. They require follow-up for longer periods in cohorts of patients with different degrees of pulmonary involvement for more consistent assessment of the prognosis and possible therapeutic interventions that can be made during the course of the disease or after its remission to prevent sequelae.

## CONCLUSION

Individuals who developed severe COVID-19 had various types of persistent symptoms after hospital discharge. In these patients, changes detected using spirometry and DLCO were present in approximately 80% of patients, representing possible functional impairment resulting from lung damage. Considering the high number of survivors of COVID-19, it is extremely important to characterize these symptoms, their persistence, and/or future remission to implement therapeutic interventions aimed at their prevention and/or treatment. Our study indicates an important economic consequence of the disease at an early post-hospital phase, given the persistent pulmonary impairment and functional capacity, limiting patients' return to routine work activities.

## AUTHOR CONTRIBUTIONS

Polese J, SanťAna L, Moulaz IR, Lara IC, Bernardi JM, Lima MD, Turini EAS, Silveira GC, Duarte S and Mill JG were responsible for the Conception and design of the work, data collection, analysis and interpretation, manuscript writing, critical review and final approval of the version to be published.

## Figures and Tables

**Table 1:** Patients profile.

	n=41
Age	
Mean/years (IQR)	51 (14)
Distribution-n (%)	
18-49 years	15 (36)
50-69 years	26 (64)
Sex-n (%)	
Male	30 (73)
Female	11 (27)
Education level-n (%)	
Illiterate	1 (2)
Up to 8 years	18 (44)
8-12 years	15 (37)
Over 12 years	7 (17)
Previous comorbidities-n (%)	30 (73)
Systemic arterial hypertension	21 (51)
Diabetes mellitus	15 (36)
Depression	4 (9)
Anxiety	6 (14)
Insomnia	9 (21)
Continuous use of medications-n (%)	30 (73)
History of smoking-n (%)	
Smokers	0 (0)
Ex-smokers	6 (14)
Never smoked	35 (85)
Alcoholism-n (%)	7 (17)
Sedentary lifestyle*-n (%)	26 (63)

IQR: interquartile range.

* defined as physical exercise less than three times a week.

**Table 2:** Tomographic analysis of the chest.

	n=20
HRCT chest	n (%)
Classical/probable COVID-19	19 (95)
Predominant pattern: bilateral, basal, ground-glass opacities/mosaic paving/peripheral consolidation/inverse halo/perilobular pattern	
Not suggestive of COVID-19	1 (5)
Lobar pneumonia/cavitation/budding tree/centrilobular nodules/lymphadenopathy/strokes/other patterns	
Disease distribution	
Bilateral	19 (95)
Lobar	1 (5)
Diffuse	14 (70)
Extension of pulmonary involvement	
26-50%	11 (55)
51-75%	8 (50)
Not measured	1 (5)
Imaging findings	
Ground-glass	19 (95)
Consolidation	14 (70)
Mosaic floor	3 (15)
Emphysema	1 (5)
Septal thickening	14 (70)
Other	4 (20)
Uncommon findings	
Pleural effusion	2 (10)

HRCT: High-resolution computed tomography.

**Table 3:** Radiographic analysis.

	n=16
Chest X-ray	n (%)
Classical/probable COVID-19	15 (93)
Multiple opacities predominantly peripheral and concentrated in lower lobes, bilaterally	
Indeterminate for COVID-19	1 (6)
Does not fit the classical findings/non-characteristic findings of COVID-19	
Disease classification	
Mild	4 (25)
Moderate	2 (12)
Severe	7 (18)
Not measured	3 (18)
